# Supplementary material for: A giant NLR gene confers broad-spectrum resistance to Phytophthora sojae in soybean
Source: Nat Commun. 2021 Nov 5;12:6263. doi: 10.1038/s41467-021-26554-8 (PMC8571336; doi:10.1038/s41467-021-26554-8)
Supplement: Supplementary file 1 — Supplementary Information [file 41467_2021_26554_MOESM1_ESM.pdf]

A giant NLR gene confers broad-spectrum resistance to *Phytophthora sojae* in soybean

Wang *et al.*

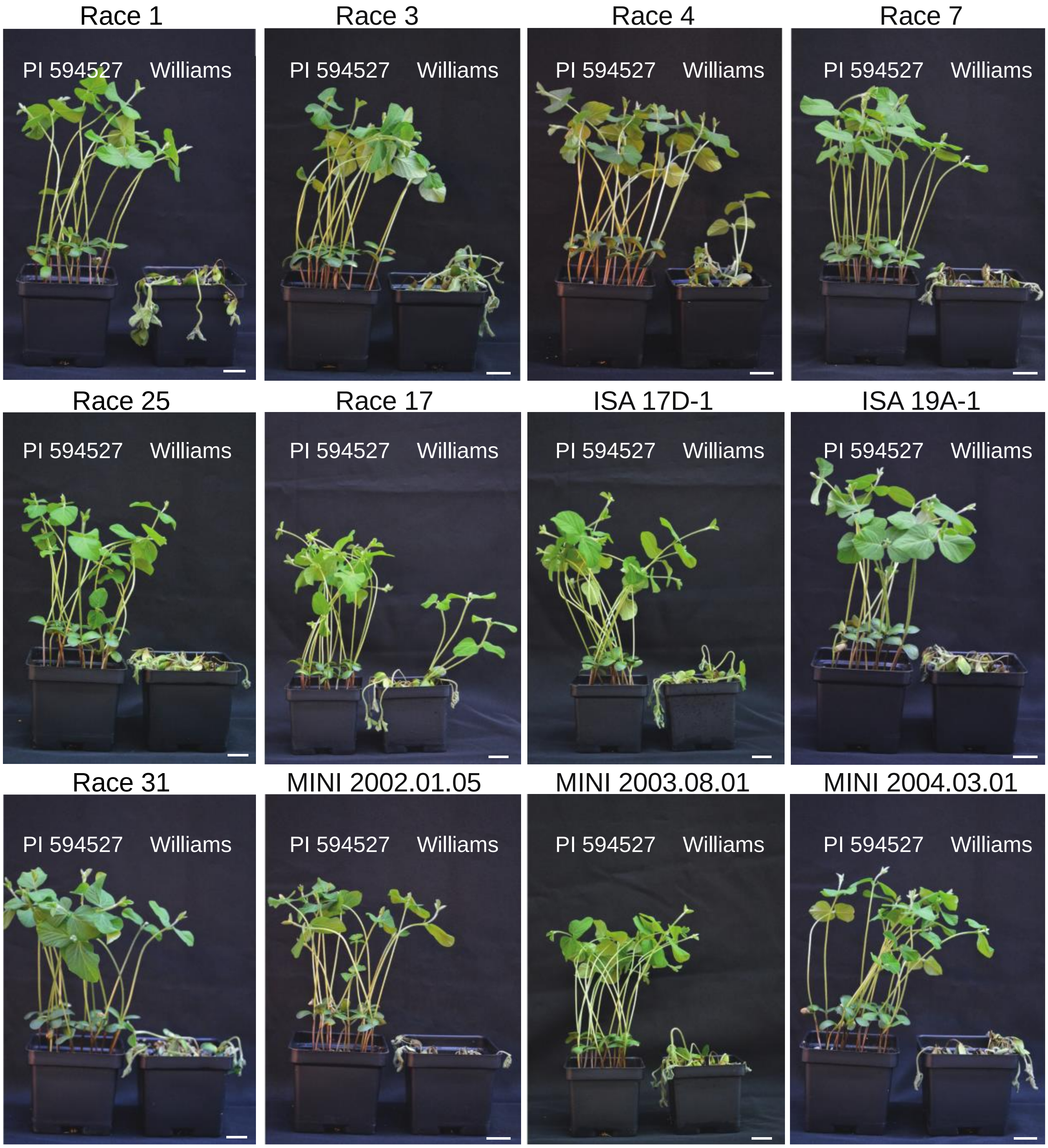

Supplementary Fig. 1. Resistance spectrum of the *Rps11* donor line. The *Rps11* donor line, PI594527 (left), and the susceptible control line, Williams (right), were inoculated with 12 races of *P. sojae*. Photos were taken 7 days after inoculation. Scale bars = 2.5 cm.

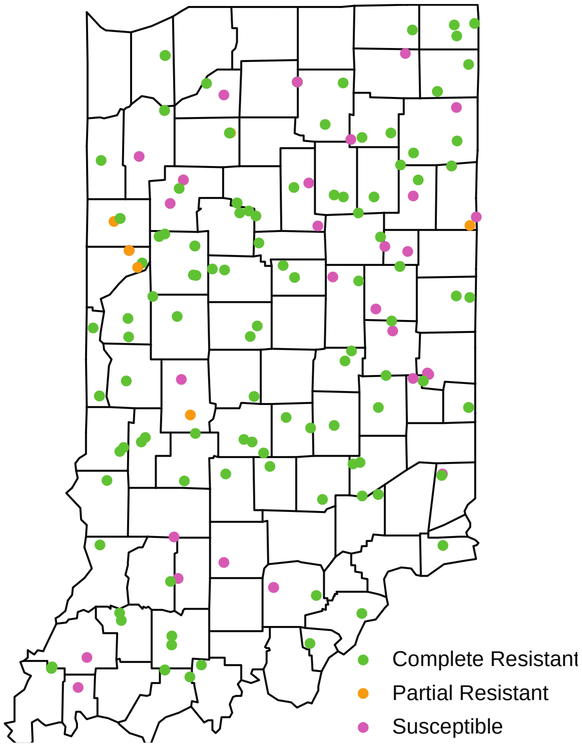

Supplementary Fig. 2. Geographic distribution of the 158 races. Green dots indicate the races that the *Rps11* locus is completely resistant to; Orange dots indicate the races that the *Rps11* locus is partially resistant to; Purple dots indicate the isolates that the *Rps11* locus is susceptible to.

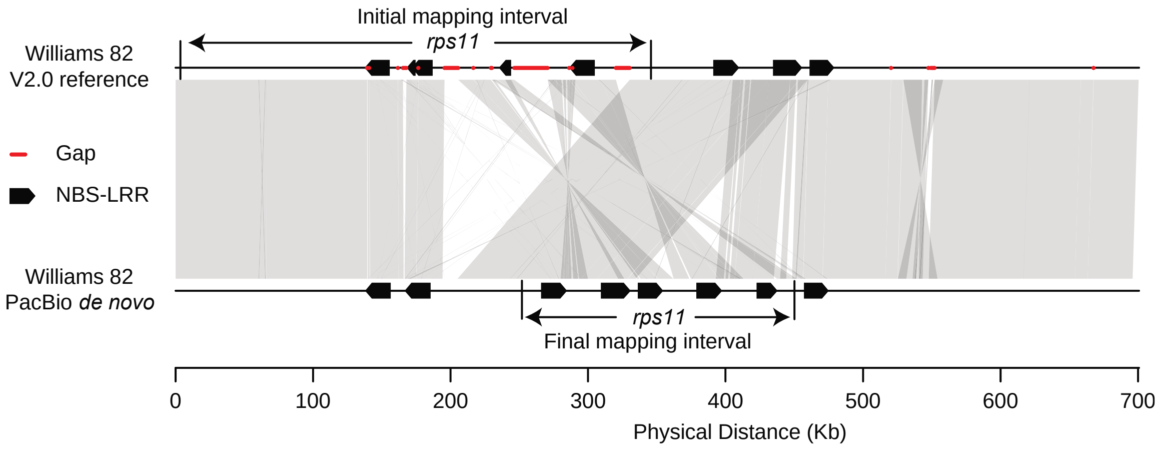

Supplementary Fig. 3. Sequence comparison between Williams 82 assembly v2.0 and the assembly v3.0. Black boxes represent NBS-LRR genes and grey shades represent syntenic blocks between two versions. Red lines/dots represent sequencing gaps in the assembly v2.0.

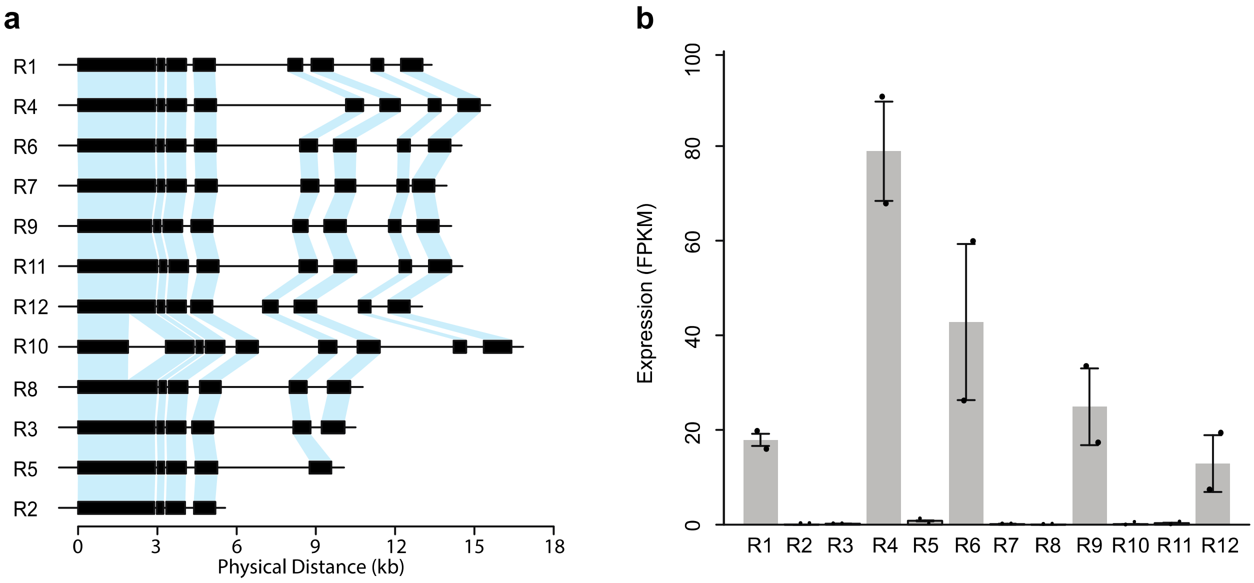

Supplementary Fig. 4. Gene models and expression pattern of the NBS-LRR genes in the *Rps11* region. a, Gene models and alignments among twelve NBS-LRR genes in PI 594527. Black boxes represent exons of each NBS-LRR gene. Light-blue shades represent alignments. **b,** Expression profile of the twelve NBS-LRR genes in the *Rps11* region (n=2 biologically independent samples). Data are presented as mean values ± SEM. *y*-axis is the expression level (FPKM) based on RNA-seq data. The error bars represent standard errors. Source data are provided as a Source Data file.

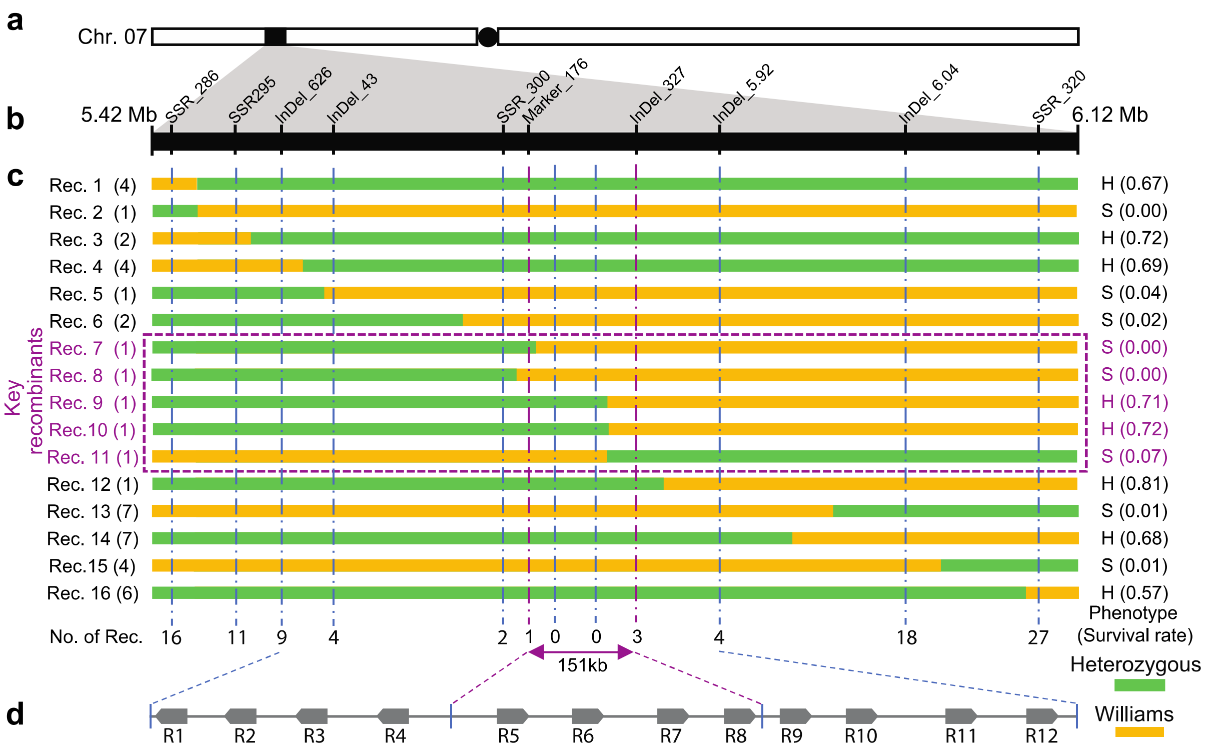

Supplementary Fig. 5. Fine mapping of the *Rps11* locus. a, Physical position of the *Rps11* locus on chromosome 7. **b,** The markers used for fine mapping. **c,** Genotype and phenotype of the recombinants. The numbers in parentheses at left side are the number of recombinants with same haplotype. Green bars represent heterozygous genotype. Orange bars represent Williams genotype. Phenotype (survival rate after inoculation) of each recombinant type are shown at right side. H means heterozygous, S means susceptible. Numbers under each dashed line are the number of recombinants at each marker. **d,** NBS-LRR gene cluster in the *Rps11* region.

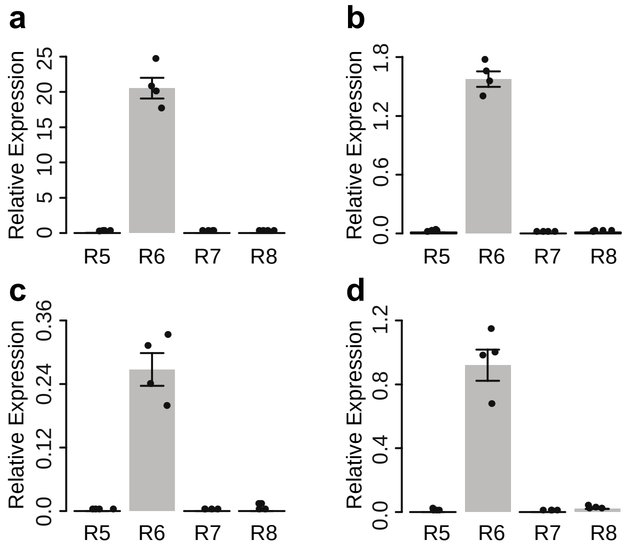

Supplementary Fig. 6. Expression levels of R5, R6, R7 and R8 in uninoculated and inoculated stems. **a,** Expression of R5, R6, R7 and R8 in uninoculated stems (n=4 biologically independent samples). The error bars represent standard errors. **b,** Expression of R5, R6, R7 and R8 in stems 4 hours after inoculation with *P. sojae* Race 1 (n=4 biologically independent samples). The error bars represent standard errors. **c,** Expression of R5, R6, R7 and R8 in stems 8 hours after inoculation with *P. sojae* Race 1 (n=4 biologically independent samples). The error bars represent standard errors. **d,** Expression of R5, R6, R7 and R8 in stems 12 hours after inoculation with *P. sojae* Race 1 (n=4 biologically independent samples). The error bars represent standard errors. Source data are provided as a Source Data file.

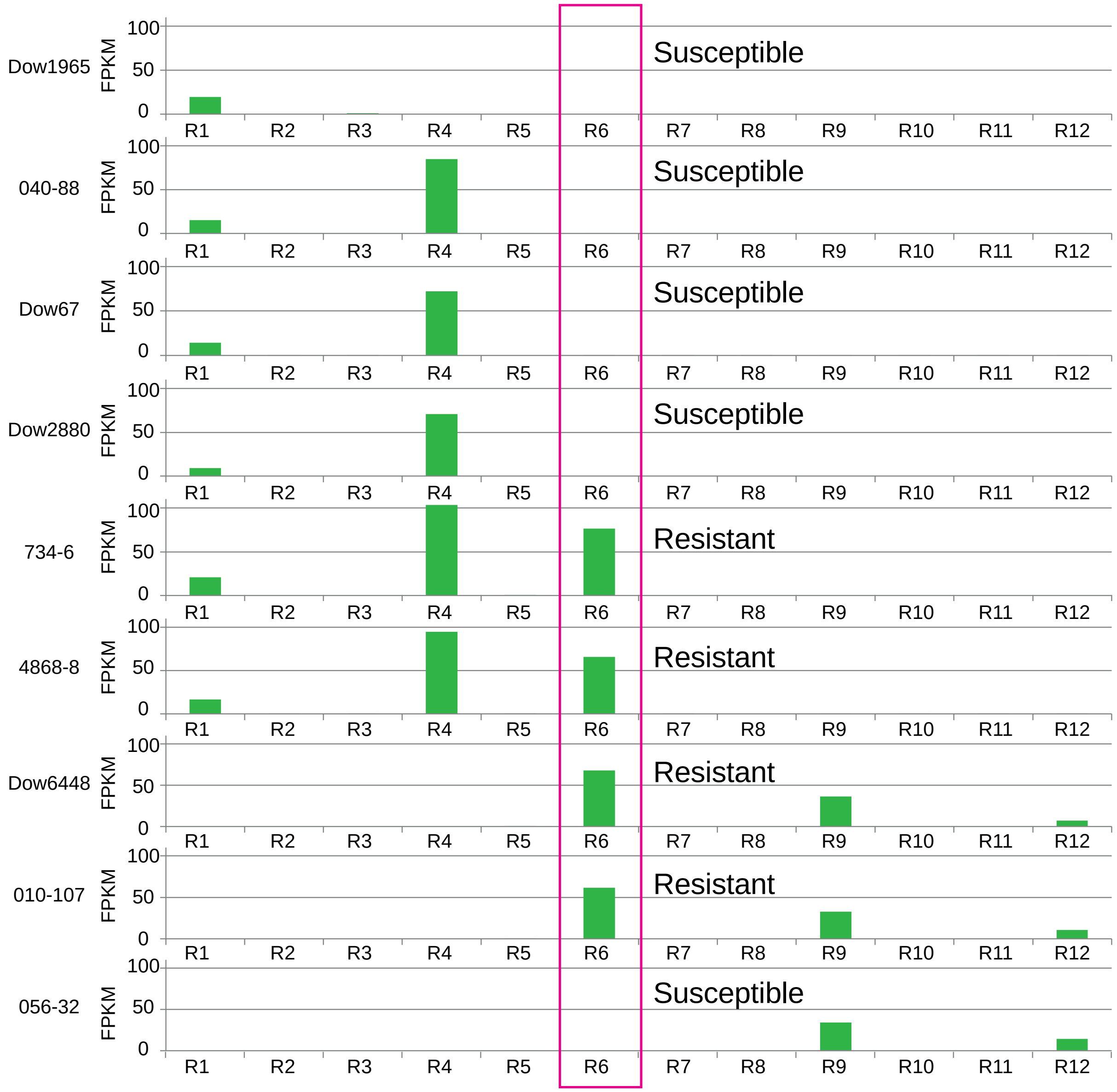

Supplementary Fig. 7. Detection of the expression of the twelve NLR genes in each key recombinant. The phenotype of each recombinant is labeled. *y*-axis is expression level (FPKM) based on RNA-seq data.

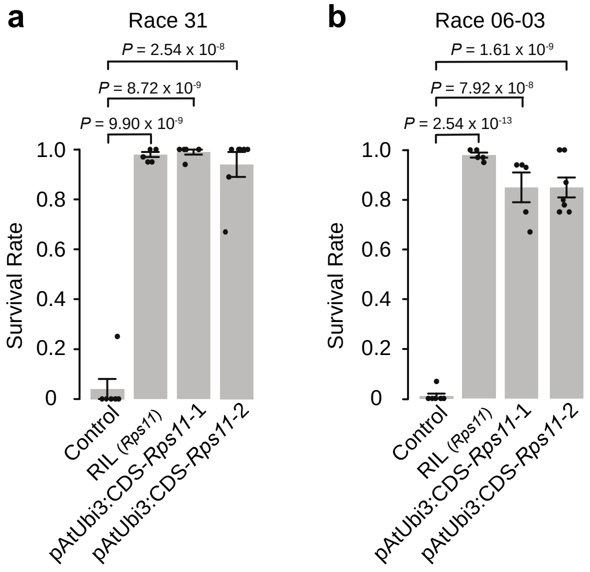

Supplementary Fig. 8. Inoculation results of the transgenic lines with race 31 and Race 06-03. a, Resistance to Race 31 of homozygous T2 families (n=5 and 7 T2 families from the two independent transgenic lines, respectively) compared with non-transgenic lines (Control, n=6 non-transgenic T2 families) and the F5 RIL (n=5 biologically independent samples). Data are presented as mean values ± SEM. *y*-axis is the survival rate after inoculation. The error bars represent standard errors. The statistical significance was determined by a two-sided *t*-test and the *P* values were shown above the plot. **b,** Resistance to Race 06-03 of homozygous T2 families (n=5 and 7 families from the two independent transgenic lines, respectively) compared with non-transgenic lines (Control, n=6 non-transgenic T2 families) and the F5 RIL (n=5 biologically independent samples). Data are presented as mean values ± SEM. *y*-axis is the survival rate after inoculation. The error bars represent standard errors. The statistical significance was determined by a two-sided *t*-test and the *P* values were shown above the plot. Source data are provided as a Source Data file.

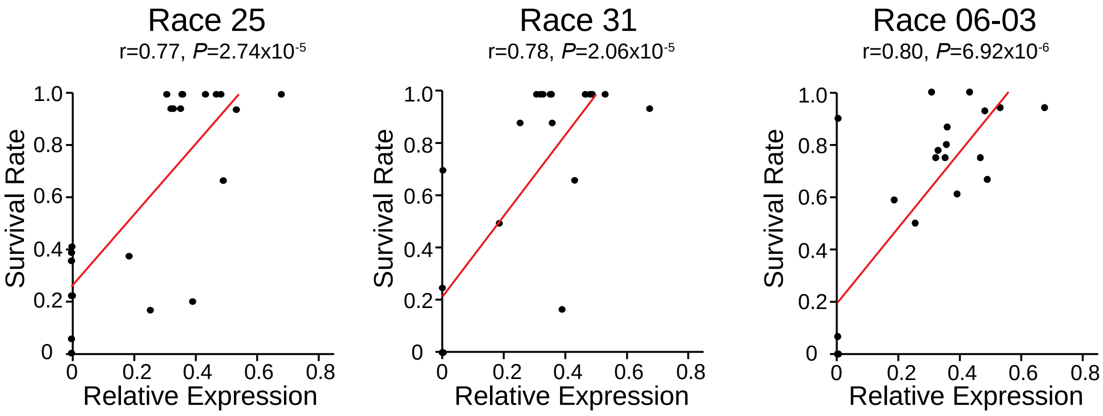

Supplementary Fig. 9. Correlation between the expression of R6 (*Rps11*) and the survival rate after inoculation in T2 population. *x*-axes are relative expression of the transgene (R6) compared to endo-reference gene. *y*-axes are survival rate after inoculation with Race 25 (left), Race 31 (middle), Race 06-03 (right). r is the Pearson correlation coefficient. The statistical significance was determined by a two-sided *t*-test and the *P* values were shown above the plot (n=22 segregating T2 families from two independent transgenic lines). Source data are provided as a Source Data file.

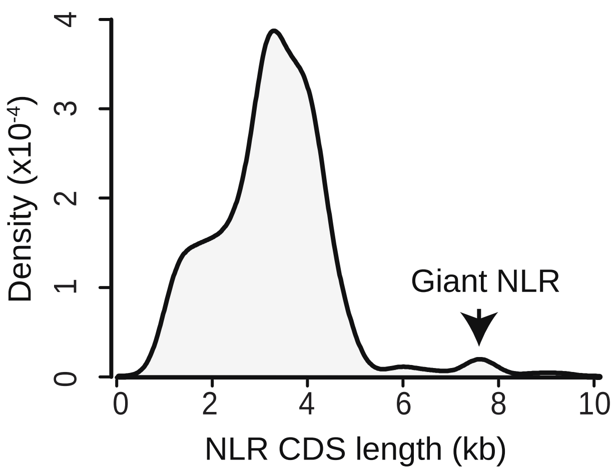

Supplementary Fig. 10. Distribution of CDS length of NLR genes in the soybean reference genome (Williams 82 v2.0). The arrow indicates the giant NLR genes.

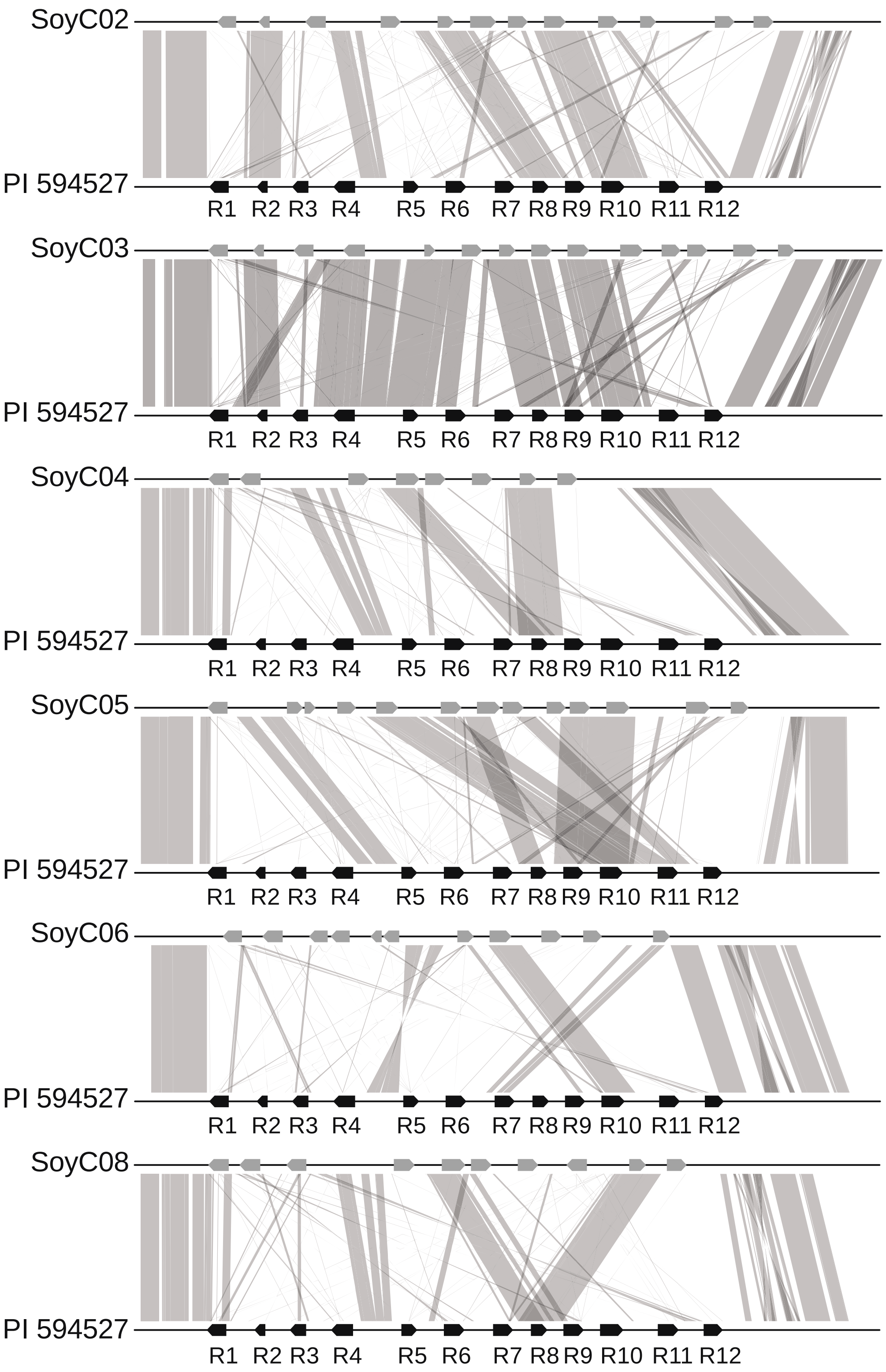
SoyC02
PI 594527
R1 R2 R3 R4 R5 R6 R7 R8 R9 R10 R11 R12
SoyC03
PI 594527
R1 R2 R3 R4 R5 R6 R7 R8 R9 R10 R11 R12
SoyC04
PI 594527
R1 R2 R3 R4 R5 R6 R7 R8 R9 R10 R11 R12
SoyC05
PI 594527
R1 R2 R3 R4 R5 R6 R7 R8 R9 R10 R11 R12
SoyC06
PI 594527
R1 R2 R3 R4 R5 R6 R7 R8 R9 R10 R11 R12
SoyC08
PI 594527
R1 R2 R3 R4 R5 R6 R7 R8 R9 R10 R11 R12

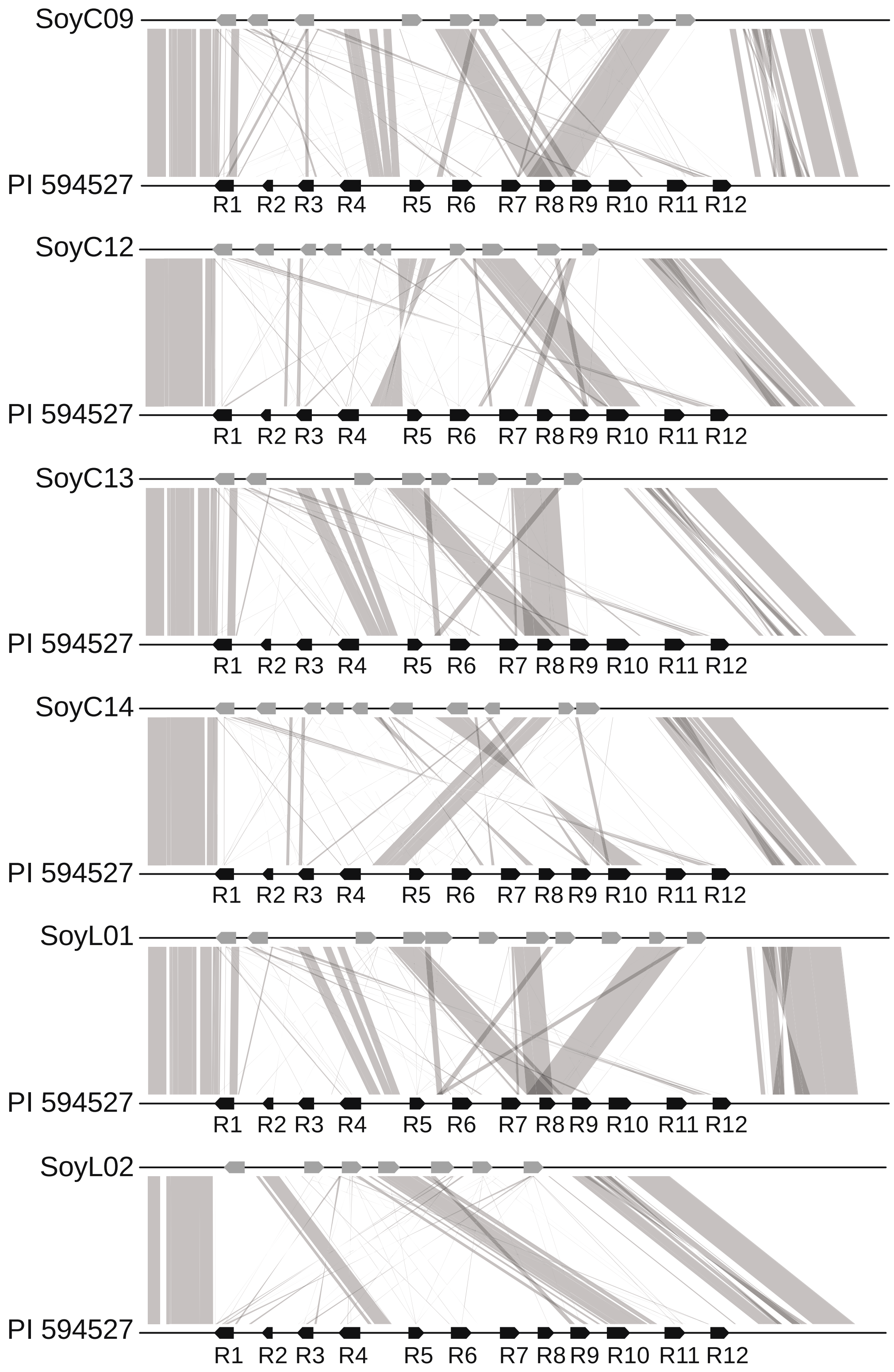
SoyC09
PI 594527
R1 R2 R3 R4 R5 R6 R7 R8 R9 R10 R11 R12
SoyC12
PI 594527
R1 R2 R3 R4 R5 R6 R7 R8 R9 R10 R11 R12
SoyC13
PI 594527
R1 R2 R3 R4 R5 R6 R7 R8 R9 R10 R11 R12
SoyC14
PI 594527
R1 R2 R3 R4 R5 R6 R7 R8 R9 R10 R11 R12
SoyL01
PI 594527
R1 R2 R3 R4 R5 R6 R7 R8 R9 R10 R11 R12
SoyL02
PI 594527
R1 R2 R3 R4 R5 R6 R7 R8 R9 R10 R11 R12

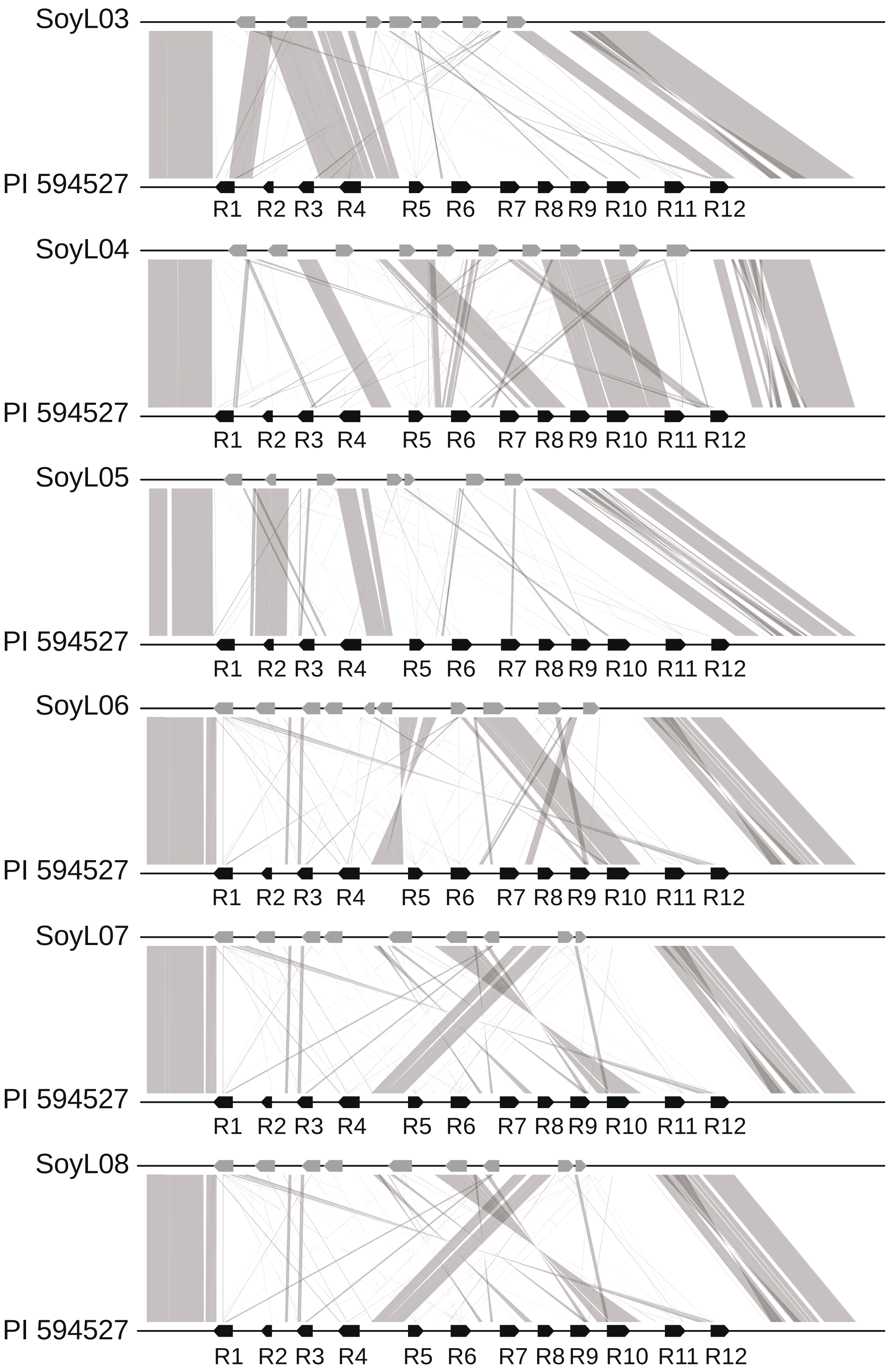
SoyL03
PI 594527
R1 R2 R3 R4 R5 R6 R7 R8 R9 R10 R11 R12
SoyL04
PI 594527
R1 R2 R3 R4 R5 R6 R7 R8 R9 R10 R11 R12
SoyL05
PI 594527
R1 R2 R3 R4 R5 R6 R7 R8 R9 R10 R11 R12
SoyL06
PI 594527
R1 R2 R3 R4 R5 R6 R7 R8 R9 R10 R11 R12
SoyL07
PI 594527
R1 R2 R3 R4 R5 R6 R7 R8 R9 R10 R11 R12
SoyL08
PI 594527
R1 R2 R3 R4 R5 R6 R7 R8 R9 R10 R11 R12

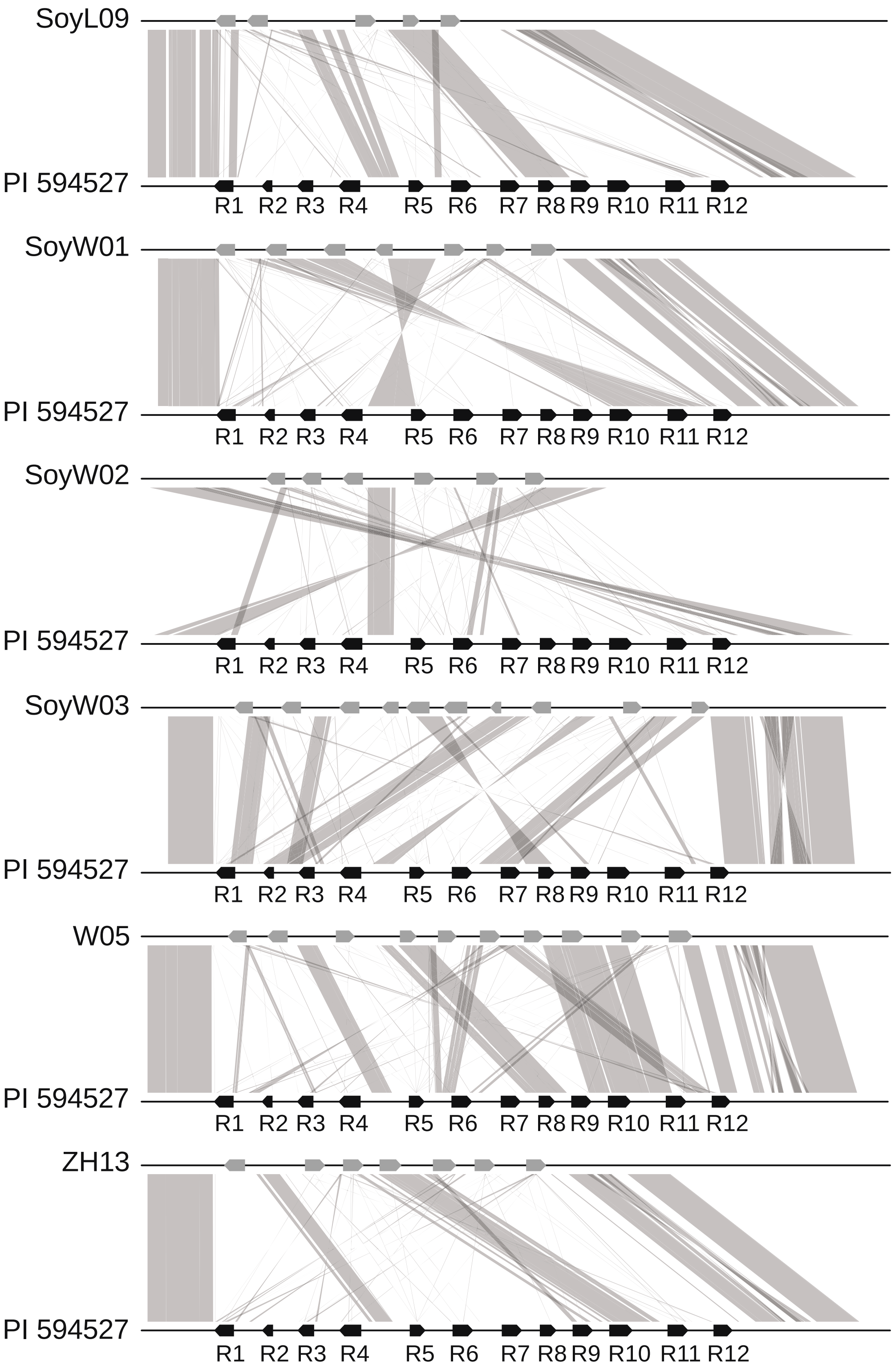
SoyL09
PI 594527
R1 R2 R3 R4 R5 R6 R7 R8 R9 R10 R11 R12
SoyW01
PI 594527
R1 R2 R3 R4 R5 R6 R7 R8 R9 R10 R11 R12
SoyW02
PI 594527
R1 R2 R3 R4 R5 R6 R7 R8 R9 R10 R11 R12
SoyW03
PI 594527
R1 R2 R3 R4 R5 R6 R7 R8 R9 R10 R11 R12
W05
PI 594527
R1 R2 R3 R4 R5 R6 R7 R8 R9 R10 R11 R12
ZH13
PI 594527
R1 R2 R3 R4 R5 R6 R7 R8 R9 R10 R11 R12

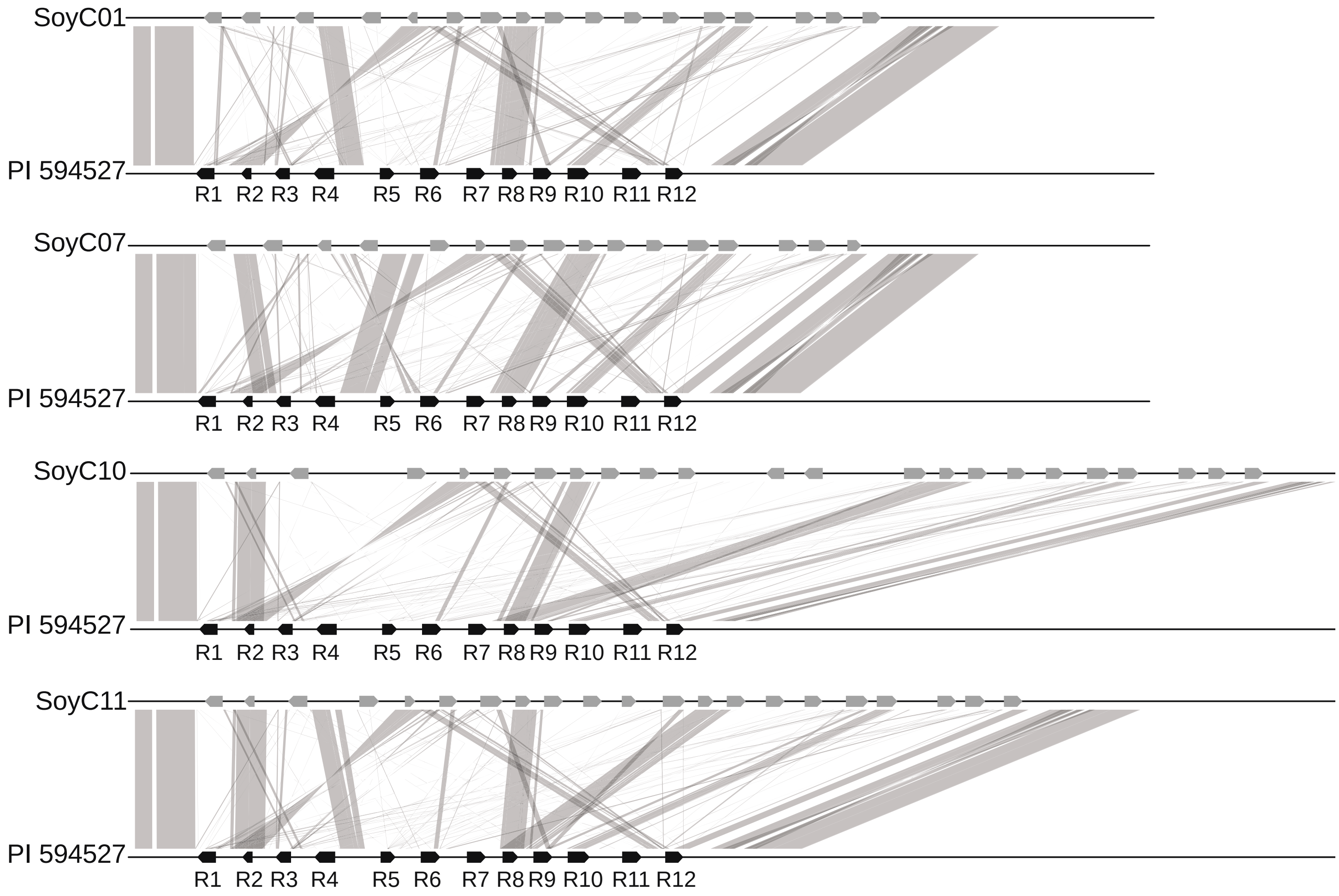

Supplementary Fig. 11. Sequence comparisons between the *Rps11* donor line and 29 diverse soybean genomes in the *Rps11* regions. Black boxes represent NLR genes from PI 594527, grey boxes represent NLR genes from other soybean genomes, and grey shade represent homologous alignments between genomes.

Supplementary Table 1. Resistance spectrum of the *Rps11* locus.

Rps11 genotype	Race1	Race4	Race7	Race25	Race3	OH001	OHC2S1	OH003	MIN1 2004.03.01	MIN1 2004.01.01	MIN1 2002.01.05	MIN1 2002.05.01	MIN1 2005.07.02	MIN1 2002.05.05
rps11	S	S	S	S	S	S	S	S	S	S	S	S	S	S
rps11	S	S	S	S	S	S	S	S	S	S	S	S	S	S
rps11	S	S	S	S	S	S	S	S	S	S	S	S	S	S
rps11	S	S	S	S	S	S	S	S	S	S	S	S	S	S
rps11	S	S	S	S	S	S	S	S	S	S	S	S	S	S
rps11	S	S	S	S	S	S	S	S	S	S	S	S	S	S
rps11	S	S	S	S	S	S	S	S	S	S	S	S	S	S
rps11	S	S	S	S	S	S	S	S	S	S	S	S	S	S
rps11	S	S	S	S	S	S	S	S	S	S	S	S	S	S
rps11	S	S	S	S	S	S	S	S	S	S	S	S	S	S
rps11	S	S	S	S	S	S	S	S	S	S	S	S	S	S
rps11	S	S	S	S	S	S	S	S	S	S	S	S	S	S
rps11	S	S	S	S	S	S	S	S	S	S	S	S	S	S
rps11	S	S	S	S	S	S	S	S	S	S	S	S	S	S
Rps11	R	R	R	R	R	R	R	R	R	R	R	R	R	R
Rps11	R	R	R	R	R	R	R	R	R	R	R	R	R	R
Rps11	R	R	R	R	R	R	R	R	R	R	R	R	R	R
Rps11	R	R	R	R	R	R	R	R	R	R	R	R	R	R
Rps11	R	R	R	R	R	R	R	R	R	R	R	R	R	R
Rps11	R	R	R	R	R	R	R	R	R	R	R	R	R	R
Rps11	R	R	R	R	R	R	R	R	R	R	R	R	R	R
Rps11	R	R	R	R	R	R	R	R	R	R	R	R	R	R
Rps11	R	R	R	R	R	R	R	R	R	R	R	R	R	R
Rps11	R	R	R	R	R	R	R	R	R	R	R	R	R	R
Rps11	R	R	R	R	R	R	R	R	R	R	R	R	R	R
Rps11	R	R	R	R	R	R	R	R	R	R	R	R	R	R
Rps11	R	R	R	R	R	R	R	R	R	R	R	R	R	R
Rps11	R	R	R	R	R	R	R	R	R	R	R	R	R	R

Rps11 represents PI 594527 genotype, and *rps11* represents Williams genotype.

Supplementary Table 2. Number of NBS-LRR genes on each chromosome in the PI 594527 genome assembly.

Chromosome ID	Length (bp)	No. of NLR
Chr01	58479646	32
Chr02	51823354	18
Chr03	46994370	58
Chr04	52535076	6
Chr05	44312942	3
Chr06	50373303	45
Chr07	45788982	16
Chr08	48806881	30
Chr09	50310076	22
Chr10	53640677	11
Chr11	39637742	12
Chr12	42723935	22
Chr13	46929662	28
Chr14	51027704	17
Chr15	53394395	36
Chr16	37902597	58
Chr17	42197827	2
Chr18	59935911	49
Chr19	50705441	28
Chr20	49315660	19
Total	976836181	512

Supplementary Table 3. List of the primers and sequences used for fine mapping and expression analysis.

Primer name	Primer sequences
SSR-07-286F	AAAAATCAGCACCCATCGAC
SSR-07-286R	AGCCCTGGCCTTATTTTGTT
SSR-07-295F	CTCTCCTTTCATTCCCCACA
SSR-07-295R	TTCTTGGAGCTTCGGAGGTA
InDel-626F	GAACTCCACTTAATCATCTCAC
InDel-626R	TTCACTCCGTCCTCGGCGGCG
InDel-43F	ATTTCCTAATTAAGTGAAAGTTTGAAATGTTATATTA
InDel-43R	GATTTATCACACTATCAAAGTGTATGAC
SSR-300F	TCGCAATATTGGCTACGATG
SSR-300R	CTGAAAACAAAATAAAAGAGAACAAA
Marker176F	CTCTGTCCCCACCTCTCC
Marker176R	CATGGTCAGTTTGATAGC
InDel-327F	TAAGTGATTCGTTTGAGTCCT
InDel-327R	TATGGTGTGGCTATGGAGATTG
InDel-5.92F	GCATCAACACTTGGCGCAAGC
InDel-5.92R	GGATAATGCGATAATTGTTCTAGC
InDel-6.04F	AAATATAGCACCCTTTAGAG
InDel-6.04R	AGCCTCACTCTCCACAT
SSR-320F	TTTAACTGAAAATACTCCGGCA
SSR-320R	TCATAATTTAAGAGACCAAACCGA
qRT-PCR-F	TGTGAACATTCGTAGTTGTC
qRT-PCR-R	TTCCACTGACTCACAAAAAG
GmActin11F	CGGTGGTTCTATCTTGGCATC
GmActin11R	GTCTTTCGCTTCAATAACCCTA
5’RACE-R1	CCAAACCAAAGCAATACTTCACC
5’RACE-R2	AGAAAATGGACTTGAGCTCCTC
