# Supplementary material for: A giant NLR gene confers broad-spectrum resistance to Phytophthora sojae in soybean
Source: Nat Commun. 2021 Nov 5;12:6263. doi: 10.1038/s41467-021-26554-8 (PMC8571336; doi:10.1038/s41467-021-26554-8)
Supplement: Supplementary file 3 — Description of Additional Supplementary Files [file 41467_2021_26554_MOESM3_ESM.pdf]

Description of Additional Supplementary Files

File Name: Supplementary Data 1

Description: Resistance evaluation of the *Rps11* locus to 158 isolates.

File Name: Supplementary Data 2

Description: Coding and protein sequences of Rps11 (R6).

File Name: Supplementary Data 3

Description: List of NBS-LRR genes across the PI 594527 genome.

File Name: Supplementary Data 4

Description: List of the NBS-LRR genes in the *Rps11* corresponding region across 30 soybean genomes.
