# Supplementary material for: A giant NLR gene confers broad-spectrum resistance to Phytophthora sojae in soybean
Source: Nat Commun. 2021 Nov 5;12:6263. doi: 10.1038/s41467-021-26554-8 (PMC8571336; doi:10.1038/s41467-021-26554-8)
Supplement: Supplementary file 8 — Reporting Summary [file 41467_2021_26554_MOESM8_ESM.pdf]

nature portfolio

Corresponding author(s): Jianxin Ma

Last updated by author(s): Oct 6, 2021

Reporting Summary

Nature Portfolio wishes to improve the reproducibility of the work that we publish. This form provides structure for consistency and transparency in reporting. For further information on Nature Portfolio policies, see our Editorial Policies and the Editorial Policy Checklist.

Statistics

For all statistical analyses, confirm that the following items are present in the figure legend, table legend, main text, or Methods section.

n/a	Confirmed	
☐	☒	The exact sample size (*n*) for each experimental group/condition, given as a discrete number and unit of measurement
☐	☒	A statement on whether measurements were taken from distinct samples or whether the same sample was measured repeatedly
☐	☒	The statistical test(s) used AND whether they are one- or two-sided<br>*Only common tests should be described solely by name; describe more complex techniques in the Methods section.*
☒	☐	A description of all covariates tested
☐	☒	A description of any assumptions or corrections, such as tests of normality and adjustment for multiple comparisons
☐	☒	A full description of the statistical parameters including central tendency (e.g. means) or other basic estimates (e.g. regression coefficient) AND variation (e.g. standard deviation) or associated estimates of uncertainty (e.g. confidence intervals)
☐	☒	For null hypothesis testing, the test statistic (e.g. *F*, *t*, *r*) with confidence intervals, effect sizes, degrees of freedom and *P* value noted<br>*Give P values as exact values whenever suitable.*
☒	☐	For Bayesian analysis, information on the choice of priors and Markov chain Monte Carlo settings
☒	☐	For hierarchical and complex designs, identification of the appropriate level for tests and full reporting of outcomes
☒	☐	Estimates of effect sizes (e.g. Cohen's *d*, Pearson's *r*), indicating how they were calculated

Our web collection on statistics for biologists contains articles on many of the points above.

Software and code

Policy information about availability of computer code

Data collection	sra-toolkit (v2.11.0) was used to collect public sequencing data
Data analysis	All software including Canu (v1.8), pbmm2 (v0.12.0), Genomic Consensus package (v2.3.2), Long Ranger (v2.2.2), Pilon (v1.22), Bionano Genomics Access software platform (Solve3.2.2_08222018), NLR-Annotator (no version, https://github.com/steuernb/NLR-Annotator), STAR (v2.7.9a) and MEGA (7.0) used for data analysis in the manuscript have been cited/refereneced.

For manuscripts utilizing custom algorithms or software that are central to the research but not yet described in published literature, software must be made available to editors and reviewers. We strongly encourage code deposition in a community repository (e.g. GitHub). See the Nature Portfolio guidelines for submitting code & software for further information.

Data

Policy information about availability of data

All manuscripts must include a data availability statement. This statement should provide the following information, where applicable:

- Accession codes, unique identifiers, or web links for publicly available datasets
- A description of any restrictions on data availability
- For clinical datasets or third party data, please ensure that the statement adheres to our policy

All the raw sequence data and the genome assembly of PI 594527 generated from this article have been deposited in the NCBI database under BioProject PRJNA718574 (https://www.ncbi.nlm.nih.gov/bioproject/?term=prjna718574). Other genome assemblies and RNA-seq data were generated by previous studies and are publicly available9,15-18. Source data are provided with this paper. The Rps11 donor line (PI 594527) is available from the USDA germplasm collection (https://npgsweb.ars-grin.gov/). The transgenic materials described in this article are protected by the patent specified in Competing Interests section. Corteva AgriscienceTM reserves the right to require a requester of such materials to enter into a non-disclosure agreement and a material transfer agreement, or other

common type of agreement, in order to receive the materials. The use of the materials will be limited to non-commercial research uses only. Please contact J.M. (maj@purdue.edu) or R.A. (rajat.aggarwal@corteva.com) regarding the transgenic materials, and requests will be responded within 60 days.

Field-specific reporting

Please select the one below that is the best fit for your research. If you are not sure, read the appropriate sections before making your selection.

[x] Life sciences [ ] Behavioural & social sciences [ ] Ecological, evolutionary & environmental sciences

For a reference copy of the document with all sections, see nature.com/documents/nr-reporting-summary-flat.pdf

Life sciences study design

All studies must disclose on these points even when the disclosure is negative.

Sample size	Sample size was determined by seeds availability. 12-30 seedlings were inoculated with P. sojae in each replicate to ensure that the survival rates (resistance) were well estimated.
Data exclusions	No data were excluded from the analysis
Replication	Three replicates were performed for the the inoculation of the recombinants and all three replicates were successful. One replicate was used for exploring the resistance spectrum as the negative control was inoculated the same time under exactly the same conditions for each isolate, therefore, the testing result can be assessed by the test result of the negative control. Failed tests were repeated. Multiple independent transgenic lines and T2 families were generated as biological replicates for functional validation.
Randomization	The recombinants were randomly picked and planted during inoculation in each replicate.
Blinding	Resistance test of the recombinants was performed before genotyping with additional fine mapping markers, therefore the test results were collected without knowing the genotype.

Reporting for specific materials, systems and methods

We require information from authors about some types of materials, experimental systems and methods used in many studies. Here, indicate whether each material, system or method listed is relevant to your study. If you are not sure if a list item applies to your research, read the appropriate section before selecting a response.

Materials & experimental systems

n/a	Involved in the study	
[x]	[ ]	Antibodies
[x]	[ ]	Eukaryotic cell lines
[x]	[ ]	Palaeontology and archaeology
[x]	[ ]	Animals and other organisms
[x]	[ ]	Human research participants
[x]	[ ]	Clinical data
[x]	[ ]	Dual use research of concern

Methods

n/a	Involved in the study	
[x]	[ ]	ChIP-seq
[x]	[ ]	Flow cytometry
[x]	[ ]	MRI-based neuroimaging
